# Fatal Multiorgan Dysfunction Syndrome Secondary to Cocaine Toxicity

**DOI:** 10.7759/cureus.98906

**Published:** 2025-12-10

**Authors:** Tutul Chowdhury, Anusha Akella, Aditi Parulkar, Nisha K Sapkota, Mujibur Majumder

**Affiliations:** 1 Internal Medicine, Interfaith Medical Center, Brooklyn, USA; 2 Pulmonary and Critical Care Medicine, Brookdale University Hospital Medical Center, Brooklyn, USA

**Keywords:** ards (acute respiratory distress syndrome), cocaine-induced lung injury, cocaine toxicity, continuous renal replacement therapy (crrt), ischemic organ injury, multi organ dysfunction syndrome (mods), multi-system organ failure, non-traumatic rhabdomyolysis

## Abstract

Cocaine toxicity is a well-recognized cause of multiorgan dysfunction due to its potent sympathomimetic, vasoconstrictive, and prothrombotic effects. Severe cases can result in cardiogenic shock, rhabdomyolysis, acute kidney injury (AKI), and acute hepatic failure. We describe a case of a 50-year-old female with a history of substance use disorder who presented with cocaine intoxication, unresponsive to naloxone, and required intubation. She developed distributive shock requiring triple vasopressor support and suffered recurrent cardiac arrests with temporary return of circulation. Laboratory findings showed rhabdomyolysis (creatine kinase (CK) 29,074), AKI (creatinine 5.8 mg/dL), elevated troponins (9,978 ng/L), severe metabolic acidosis, and hepatic injury. Despite continuous renal replacement therapy (CRRT), empiric broad-spectrum antibiotics, and maximal supportive care, she remained comatose with progressive multiorgan failure and expired on hospital day three. This report underscores the severe systemic and often fatal complications of cocaine toxicity.

## Introduction

Cocaine is a potent stimulant derived from the coca plant and continues to pose a significant global public health challenge because of its widespread misuse and potentially life-threatening complications [1]. Its sympathomimetic and vasoconstrictive effects can cause extensive multiorgan injury involving the cardiovascular, renal, hepatic, pulmonary, and neurological systems. The overall mortality rate in acute cases varies, with some studies documenting rates of approximately 3.8% or higher, depending on the severity of organ failure [2]. While often perceived as producing only short-lived effects, cocaine toxicity can lead to severe and irreversible damage, including rhabdomyolysis, ischemic organ injury, and cardiac arrest [2]. We present a case of fatal multiorgan failure secondary to cocaine intoxication, highlighting the severe systemic effects of this drug.

## Case presentation

A 50-year-old female with a past medical history of substance use disorder was brought into the emergency department for suspected drug intoxication. The patient was administered Narcan but did not respond and was noted to be significantly altered. She remained obtunded and required endotracheal intubation with initiation of mechanical ventilation. On admission, urine toxicology came out positive for cocaine. On presentation, vitals showed blood pressure of 100/34 mmHg with a mean arterial pressure of 57, heart rate of 100 beats per minute, and temperature of 98.7 °F. On examination, pupils were reactive to light but anisocoric, with the left pupil measuring 1 mm and the right 2-3 mm. Extremities were cool to the touch, suggesting poor perfusion. Labs on admission are presented in Table 1.

**Table 1:** Lab studies on day 1 COVID-19: coronavirus disease 2019; PCR: polymerase chain reaction

Investigation	Value	Reference range
Hemoglobin	13.6	11.0–15.0 g/dL
Hematocrit	42.9	35-46%
White blood cells	8.7	3.8–5.3 10⁶/uL
Platelets	289	130–400 10^3^/uL
Glucose	143	80–115 mg/dL
Blood urea nitrogen	64	9.8–20.1 mg/dL
Creatinine	2.1	0.57–1.11 mg/dL
Sodium	135	136–145 mmol/L
Potassium	4.8	3.5–5.1 mmol/L
Chloride	97	98–107 mmol/L
Bicarbonate	29	23–31 mmol/L
Calcium	9.1	8.8–10.0 mg/dL
Albumin	3.9	3.2–4.6 g/dL
Magnesium	2.7	1.6–2.6 mg/dL
COVID-19 PCR	Negative	Negative
Prothrombin time	13	9.8–13.4 sec
international normalised ratio (INR)	1.19	0.85–1.15
Partial thromboplastin time (PTT)	30.2	24.9–35.9 sec
Lactate	3.6	0.5–2.2 mmol/l
Creatine kinase (CK)	29074	30–223 U/L
Troponin	9978.2	5–11.8 pg/ml
Urine toxicology	Positive for cocaine	Negative

**Figure 1:**
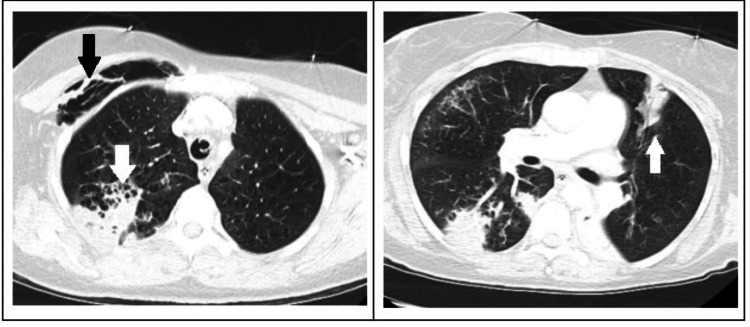
CT scan of the chest showing bilateral consolidation (both white arrows) and subcutaneous emphysema (black arrow on the left) CT: computed tomography

COVID-19 and the respiratory viral panel were negative. No leukocytosis was noted during hospitalization. CT head was unremarkable; however, CT chest showed subcutaneous emphysema and multifocal patchy consolidation in both lungs consistent with multifocal pneumonia (Figure 1). However, blood, sputum, and urine cultures all returned negative results.

**Figure 2:**
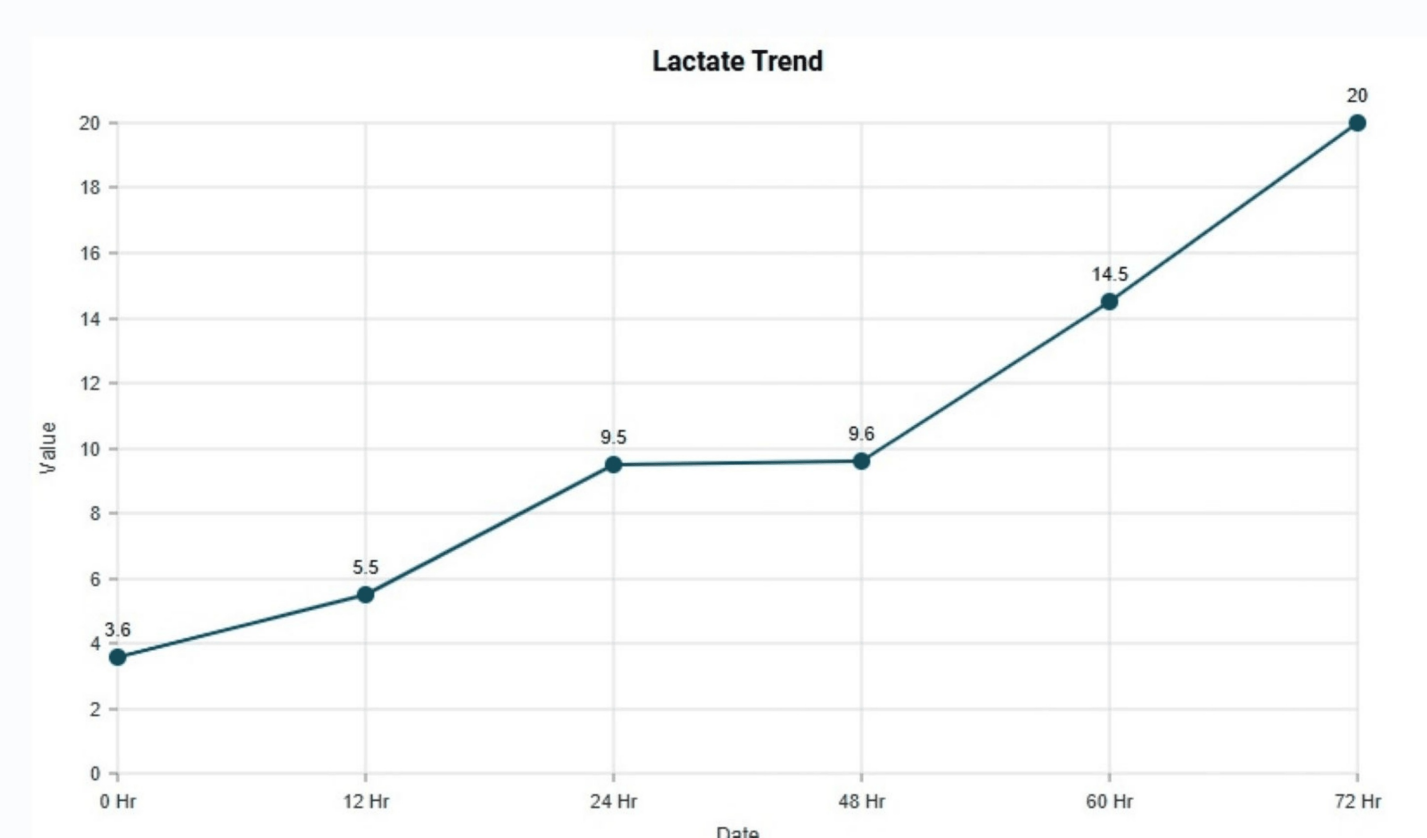
Trend of Lactate during hospitalization X-axis: time since hospitalization. Y-axis: value of lactate (mmol/L) Hr: hour

The patient was also noted to be hypotensive and needed vasopressor support with norepinephrine. The following morning, vasopressin was initiated; however, the patient developed worsening hypotension requiring escalation of epinephrine doses, which subsequently precipitated episodes of ventricular tachycardia. As her blood pressure continued to deteriorate, she experienced two cardiac arrests, one lasting approximately four minutes and the second lasting two minutes. Return of spontaneous circulation (ROSC) was achieved after each event, and a bicarbonate infusion was started. Eventually, on day two, the patient developed multiorgan failure with severe metabolic acidosis, marked electrolyte derangements, significantly elevated liver enzymes, and a lactate level >9 mmol/L. Lactate trended from 3.6 mmol/L to 20 mmol/L, suggestive of significant tissue hypoxia (Figure 2).

**Figure 3:**
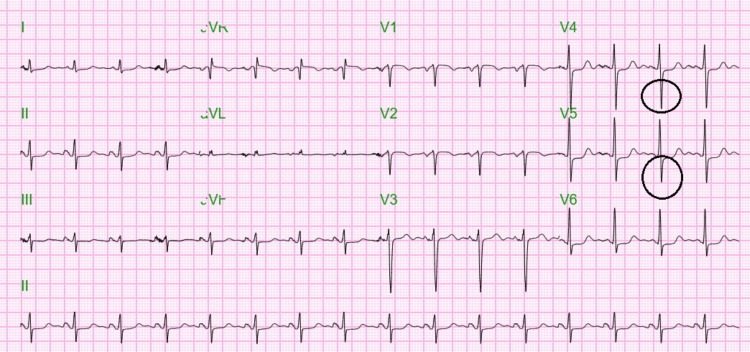
12 lead electrocardiogram showing ST segment depressions (black circles) in V4 and V5

Continuous renal replacement therapy (CRRT) was initiated. Cardiac evaluation revealed elevated troponins and ST depressions in leads V4-V6 (Figure 3), consistent with non-ST elevation myocardial infarction, for which therapy was initiated but later discontinued due to rectal bleeding.

**Figure 4:**
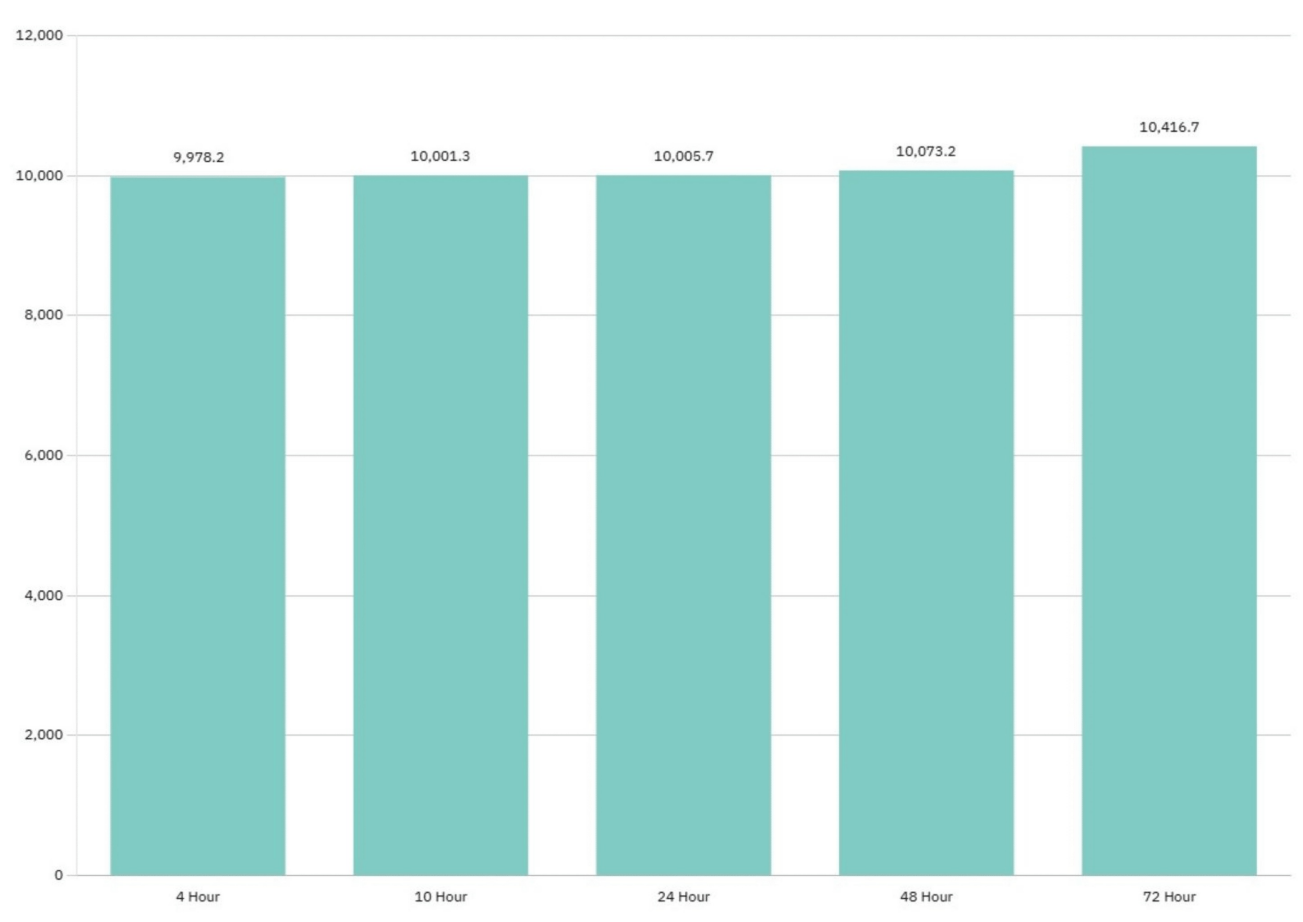
Chart demonstrating the trend of troponin during hospitalization X-axis: duration since admission in hours. Y-axis: troponin level (pg/ml)

The patient also developed acute respiratory distress syndrome (ARDS) secondary to pneumonia on day three. Lung protective ventilation was pursued. Arterial blood gas analysis showed a pH of 7.0, pCO_2_ of 62.0, pO_2_ of 87.0, and oxygen saturation of 97%. Her Horowitz Index for Lung Function was 87 mmHg, suggestive of severe ARDS. She was empirically treated with vancomycin and cefepime. Throughout her ICU stay, the patient remained comatose with no improvement in neurological function and developed progressive multiorgan failure, characterized by worsening metabolic acidosis, refractory hyperkalemia, and persistent hypoxemia despite maximal supportive therapy. Creatine Kinase (CK) level was 29,074 U/L, suggestive of rhabdomyolysis. Urinalysis was positive for blood. Troponin trended from 9978.2 pg/ml to 10416.7 pg/ml, revealing cardiac injury (Figure 4).

Creatinine was elevated persistently, peaking at 5.8 mg/dL, suggestive of AKI. On day three of hospitalization, the patient went into cardiac arrest for the third time and expired after 30 minutes of cardiopulmonary resuscitation efforts.

## Discussion

Cocaine exerts its toxic effects through several mechanisms. It inhibits the neuronal reuptake of norepinephrine, leading to excessive activation of adrenergic receptors, thereby causing vasospasm and an exaggerated sympathetic response [1,2]. It also acts as a sodium channel blocker, giving it local anesthetic properties. Cocaine use can lead to ischemic injury through cardiogenic shock or intense vasospasm of vessels supplying vital organs. Activation of alpha-adrenergic receptors in coronary arteries may produce coronary vasospasm and vasospastic angina, as likely occurred in this patient before cardiac arrest. It may also promote thromboembolic events, leading to multiorgan ischemia [3].

Multiorgan injury or multiorgan dysfunction syndrome from cocaine use is well documented. Our patient developed rhabdomyolysis with markedly elevated creatine phosphokinase (CPK) levels, resulting in AKI. Cocaine-related nephrotoxicity can occur through multiple pathways, including rhabdomyolysis, thrombotic microangiopathy, vasculitis, acute interstitial nephritis, or renal infarction [4]. Cocaine-induced hepatocellular injury results either from cytochrome P450 metabolism producing the toxic metabolite norcocaine or from hepatic ischemia caused by vasoconstriction [5]. It presents rapidly within a week, with aspartate aminotransferase (AST) levels exceeding alanine aminotransferase (ALT) by 10-12 times the upper limit of normal and elevated lactate dehydrogenase (LDH), yielding an ALT/LDH ratio near 0.5. Rhabdomyolysis and hyperthermia often coexist. Management is primarily supportive, and N-acetylcysteine (NAC) may be beneficial [5,6].

Pulmonary toxicity from smoking crack cocaine, termed “crack lung,” involves diffuse alveolar damage and hemorrhagic alveolitis within 48 hours of exposure [7]. It may present as diffuse alveolar hemorrhage or pulmonary eosinophilia and sometimes resembles interstitial lung disease [6][7]. Vasculitis associated with adulterated cocaine further broadens the spectrum of organ injury.

## Conclusions

This report highlights the multisystemic toxic effects of cocaine, including hepatic, renal, muscular, and pulmonary injury. The patient’s presentation underscores cocaine’s potential to cause acute hepatocellular necrosis through both direct metabolic toxicity and ischemic mechanisms, compounded by rhabdomyolysis-induced renal impairment and pulmonary injury consistent with “crack lung.” Recognizing these overlapping pathophysiologic pathways is essential for timely diagnosis and management. Supportive care remains the cornerstone of therapy. This report reinforces the importance of maintaining a high index of suspicion for cocaine-related multiorgan injury in patients presenting with unexplained systemic failure. Fatal multiorgan dysfunction syndrome is often an irreversible consequence of cocaine use. This severe condition highlights that no amount of cocaine is safe, as it can lead to catastrophic organ failure in vulnerable individuals.
